# Historical developments, hotspots, and trends in tardive dyskinesia research: a scientometric analysis of 54 years of publications

**DOI:** 10.3389/fpsyt.2023.1194222

**Published:** 2023-06-02

**Authors:** Anuradha Baminiwatta, Christoph U. Correll

**Affiliations:** ^1^Department of Psychiatry, Faculty of Medicine, University of Kelaniya, Ragama, Sri Lanka; ^2^Department of Psychiatry and Molecular Medicine, Donald and Barbara Zucker School of Medicine at Hofstra/Northwell, Hempstead, NY, United States; ^3^Department of Psychiatry, Zucker Hillside Hospital, Glen Oaks, NY, United States; ^4^Department of Child and Adolescent Psychiatry Charité Universitätsmedizin, Berlin, Germany

**Keywords:** tardive dyskinesia, bibliometrics, scientometric analysis, trends, antipsychotics

## Abstract

**Background:**

Since being recognized as an important drug-induced clinical entity during the 1960s, tardive dyskinesia (TD) has generated an extensive body of research seeking to understand its clinical characteristics, epidemiology, pathophysiology and management. Modern scientometric approaches allow interactive visualization of large bodies of literature to identify trends and hotspots within knowledge domains. This study thus aimed to provide a comprehensive scientometric review of the TD literature.

**Methods:**

Web of Science was searched for articles, reviews, editorials and letters with the term “tardive dyskinesia” in the title, abstract, or keywords through 12/31/2021. A total of 5,228 publications and 182,052 citations were included. Annual research output, prominent research areas, authors, affiliations and countries were summarized. VOSViewer and CiteSpace were used for bibliometric mapping and co-citation analysis. Structural and temporal metrics were used to identify key publications in the network.

**Results:**

TD-related publications peaked in the 1990s, gradually declined after 2004, and showed a further small increase after 2015. The most prolific authors were Kane JM, Lieberman JA, and Jeste DV overall (1968–2021), and Zhang XY, Correll CU and Remington G in the last decade (2012–2021). The most prolific journal was the Journal of Clinical Psychiatry overall, and the Journal of Psychopharmacology in the last decade. Knowledge clusters in the 1960–1970s dealt with clinical and pharmacological characterization of TD. In the 1980s, epidemiology, clinical TD assessment, cognitive dysfunction and animal models predominated. During the 1990s, research diverged into pathophysiological studies, especially oxidative stress, and clinical trials on atypical antipsychotics, with a focus on clozapine and bipolar disorder. In the 1990–2000s, pharmacogenetics emerged. More recent clusters include serotonergic receptors, dopamine-supersensitivity psychosis, primary motor abnormalities of schizophrenia, epidemiology/meta-analyses, and advances in TD treatment, particularly vesicular monoamine transporter-2 inhibitors since 2017.

**Conclusion:**

This scientometric review visualized the evolution of scientific knowledge on TD over more than five decades. These findings will be useful for researchers to find relevant literature when writing scientific articles, choosing appropriate journals, finding collaborators or mentors for research, and to understand the historical developments and emerging trends in TD research.

## Background

Tardive dyskinesia (TD) is a drug-induced movement disorder that has been, for many decades, an unrelenting concern for psychiatrists and a burden to patients, owing to its potentially irreversible nature (1). Definition of this syndrome has evolved over the years, and it is defined in the latest edition of the Diagnostic and Statistical Manual of Mental Disorders (DSM-5) as “involuntary athetoid or choreiform movements, generally involving the tongue, lower face and jaw, and extremities, associated with long-term use of dopamine receptor blocking agents” (2). Perioral movements associated with conventional antipsychotics were reported as far back as in the 1950s (3), before the term TD was coined by Faurbye and colleagues in 1964 (4). Since then, scientific interest and investigation into TD have progressed remarkably, broadening and deepening the understanding of its clinical characterization, epidemiology, pathophysiology and management (5). Despite the development of second-generation, so-called “atypical” antipsychotics, which have less parkinsonian adverse effects than first-generation, so-called “typical antipsychotics” (6), TD has not disappeared, as patient and treatment risk factors continue (7) and as all postsynaptic dopamine antagonists/partial agonists have been associated with at least some TD risk (8, 9).

Since present thinking and future directions in any scientific domain are founded upon insights gained through its history, it is of educational, epistemological and scientific value to the practicing clinical psychiatrists and researchers to have a broad reflective outlook on the historical developments and trends within the published literature on TD. Bibliometrics, originally defined by Pritchard (10) as “the application of mathematical and statistical methods to books and other communication media,” provides a useful framework for the study of patterns and trends within a large body of literature. Advancement of statistical methods and computing has produced more sophisticated bibliometric tools that utilize computer algorithms to detect and map trends and hotspots in knowledge domains (11). The application of bibliometrics and systematic mapping to study science research has been termed ‘scientometrics’ (12). Although narrative reviews have previously attempted to describe the history of TD research (1, 5), their scope and objectivity have been limited. To the best of our knowledge, a review of the TD literature informed by bibliometric indices has not been attempted so far. However, this approach is valuable, as it can provide a more comprehensive and objective analysis of how scientific knowledge on TD has progressed over the years, and it also has the potential to reveal emerging trends. This study thus reviewed the historical developments, hotspots and trends in TD literature, guided by bibliometrics. We hypothesized that certain knowledge areas and scientific themes would emerge and not be constant over time.

## Methods

### Data retrieval

The Web of Science (WoS) was used to access bibliographic data indexed in Science Citation Index Expanded (SCIE). SCIE indexes over 9,200 journals across 178 scientific disciplines, with more than 53 million records and 1.18 billion cited references dating back to 1900 (13). The availability of citation data makes the WoS data conducive for document co-citation analysis; WoS is also the primary data source for CiteSpace. WoS SCIE database was searched for all documents with “tardive dyskinesia” as a topic from 1900 to 31st of December, 2021; this identified all documents where the term “tardive dyskinesia” was mentioned in the title, abstract or keywords. Only *articles, reviews, editorial material* and *letters* were included; other document types such as *meeting abstracts*, *proceedings papers* and *book reviews,* were excluded. Bibliographic data were exported as ‘full records with cited references’. The literature search methods used in this study were similar to those used in many previous scientometric studies (14–17).

### Scientometric analysis and visualization

Scientometric analyses were performed using three analytical tools – (1) the in-built Analysis tool on WoS; (2) VOSviewer (version 1.6.16); and (3) CiteSpace (version 6.1.R4).

#### WoS analysis

Summary information about the most prolific authors, countries, affiliations, journals and annual publication trends were obtained from WoS Analysis tool. The findings from the entire study period and the last decade (2012–2021) were tabulated side-by-side to explore recent trends. For author names, alternative names (e.g., Kane J and Kane JM) were merged. For institutions, WoS has conducted a unification process over the past few years, where institution’s variants and parent/child relationships are mapped and linked to a preferred name.

#### VOSviewer

VOSviewer is a software used for creating network maps based on bibliographic data (18). Keyword co-occurrence networks and country-collaboration networks based on co-authorship were visualized using VOSviewer. In VOSviewer, a *cluster* is a set of items (e.g., countries, keywords) that are closely related on a map and are visualized with the same color. In VOSviewer maps, items with greater importance (e.g., higher frequency of co-occurrence for keywords) are shown with larger labels and larger circles. A *link* is a connection between two items, e.g., co-authorship links between countries and co-occurrence links between keywords. Each link is assigned a numerical value called *link strength,* which is represented by the thickness of the line connecting two items.

#### CiteSpace

CiteSpace has been developed for visualizing transient patterns and emerging trends in a knowledge domain using co-citation analysis (11). Co-citation analysis draws from earlier methods proposed by Small (19): Co-citation is the frequency of two documents being cited together; the more co-cited two documents are, the more likely they are semantically related. CiteSpace employs computer algorithms to map the co-citation network and to automatically label clusters using keywords used in citing articles by employing a log-likelihood ratio (LLR) algorithm. CiteSpace provides metrics of structural and temporal properties of nodes and the network. Structural metrics include *centrality*, an indicator of the extent to which a node lies in the middle of a path connecting different nodes, based on concepts of Freeman (20). Within structural metrics, high-centrality nodes represent pivotal points within the literature (11). Temporal metrics include citation burstness, which is detected using algorithms proposed by Kleinberg (21), and indicate rapid surges of interest within the scientific community toward a particular node at a particular period of time. *Sigma* is an indicator of how influential a node is based on both structural and temporal metrics; sigma is computed as (centrality+1)^burstness^. In presenting citation bursts, similar to the approach taken by Sabe et al. (2022), irrelevant citations, such as those referring to classification of mental disorders (e.g., DSM), were excluded (22). The quality of network clustering configurations is indicated by the silhouette value, and the clarity of network decomposition by the modularity Q. The Q metric ranges from 0 to +1 and values greater than 0.3 are considered significant. Silhouette value ranges from −1 to +1, and when coefficients exceed 0.3, 0.5, or 0.7, the network is considered homogenous, reasonable, or highly credible, respectively.

## Results

### Publication and citation trends over the years (1968–2021)

**Figure 1:**
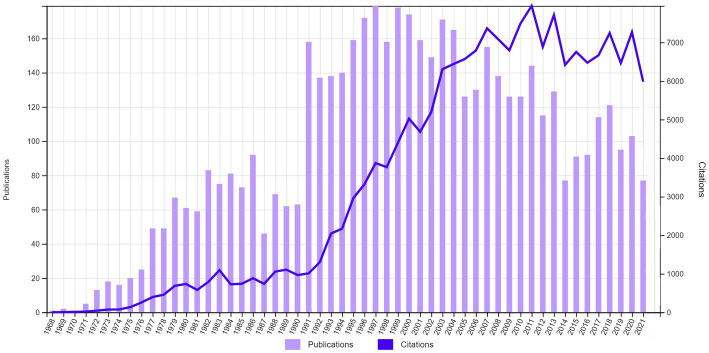
Trends in the annual research output and citations related to tardive dyskinesia (1968–2021).

A total of 5,228 publications on the topic of TD were identified from WoS between 1900 and 12/31/2021. There had been a gradual increase in publications from the early 1970s to 1986, followed by a small trough during 1987–1990 (Figure 1). The greatest surge of publications was observed during the 1990s, which peaked in 1997 (179 publications) and continued into the early 2000s. From 2004 to 2014 there had been a gradual decline in the publication output; however, since 2015 there has been another brief increase in publications.

The annual number of citations for TD-related publications rose steeply from the early 1990s and plateaued between 2007 and 2020, with a peak in 2011 (Figure 1). Altogether, there were a total of 182,052 citations for the 5,228 publications, resulting in an average of 34.8 citations per publication and an h-index of 163.

### Most active research areas

*Psychiatry, neurosciences/neurology, pharmacology/pharmacy, psychology* and *general internal medicine* were the five most active research areas in TD, in that order, during the total study period (1968–2021); the same research areas were prominent during the last decade (2012–2021), but *general internal medicine* has overtaken *psychology* in the order of importance (Table 1). A decline in the contribution from *psychiatry* (from 58.7 to 44.8%) and *psychology* (8.8 to 5%) occurred. Other trends of note include a rise in contributions from *Biochemistry & Molecular Biology* (from 2.5 to 4%), *Pediatrics* (from 1.3 to 1.8%) and *Science Technology Other Topics* (from 0.65 to 2%).

**Table 1:** Most active research areas during the entire study period (1968–2021) and the last decade (2012–2021).

1968–2021			2012–2021		
Research area	Number of publications	%	Research area	Number of publications	%
Psychiatry	3,067	58.665	Psychiatry	444	44.758
Neurosciences Neurology	1960	37.490	Neurosciences Neurology	394	39.718
Pharmacology Pharmacy	1,668	31.905	Pharmacology Pharmacy	333	33.569
Psychology	463	8.856	General Internal Medicine	57	5.746
General Internal Medicine	333	6.370	Psychology	50	5.040
Biochemistry Molecular Biology	134	2.563	Biochemistry Molecular Biology	40	4.032
Behavioral Sciences	112	2.142	Behavioral Sciences	30	3.024
Genetics Heredity	87	1.664	Science Technology Other Topics	20	2.016
Geriatrics Gerontology	87	1.664	Research Experimental Medicine	19	1.915
Research Experimental Medicine	77	1.473	Pediatrics	18	1.815
Public Environmental Occupational Health	73	1.396	Health Care Sciences Services	17	1.714
Pediatrics	69	1.320	Genetics Heredity	16	1.613
Toxicology	66	1.262	Chemistry	14	1.411
Health Care Sciences Services	49	0.937	Geriatrics Gerontology	13	1.310
Endocrinology Metabolism	42	0.803	Toxicology	13	1.310
Surgery	37	0.708	Nursing	11	1.109
Chemistry	34	0.650	Endocrinology Metabolism	10	1.008
Science Technology Other Topics	34	0.650	Public Environmental Occupational Health	9	0.907
Cell Biology	24	0.459	Gastroenterology Hepatology	6	0.605
Dentistry Oral Surgery Medicine	22	0.421	Biotechnology Applied Microbiology	5	0.504
Nursing	19	0.363	Dentistry Oral Surgery Medicine	5	0.504
Biotechnology Applied Microbiology	16	0.306	Physiology	5	0.504
Nutrition Dietetics	10	0.191	Surgery	5	0.504
Physiology	10	0.191	Food Science Technology	4	0.403
Life Sciences Biomedicine Other Topics	9	0.172	Nutrition Dietetics	4	0.403

The sum of percentages exceeds 100% since one publication can belong to more than one research area.

### Most prolific authors

The 25 most prolific authors in TD research during the whole study period and the last decade are listed separately in Table 2. With 101 publications spanning over 4 decades, Kane JM was the most prolific author overall, followed by Lieberman JA and Jeste DV. Kane JM has been most active in TD research during the 1980s. The three most prominent authors during the last decade were Zhang XY, Correll CU and Remington G.

**Table 2:** Most prolific authors in TD publications during the entire study period (1968–2021) and the last decade (2012–2021).

1968–2021		2012–2021	
Author	Number of publications	Author	Number of publications
Kane JM	101	Zhang XY	31
Lieberman JA	96	Correll CU	28
Jeste DV	88	Remington G	25
Meltzer HY	64	Tan YL	24
Casey DE	64	Jankovic J	22
Remington G	64	Iyo M	20
Kennedy JL	59	Yang FD	20
Zhang XY	50	Kane JM	19
Van Harten PN	49	Kanahara N	18
Gerlach J	45	Meltzer HY	18
Waddington JL	42	Van Harten PN	18
Wyatt RJ	42	Caroff SN	17
Correll CU	41	Kennedy JL	17
Jankovic J	41	Chen DC	16
Tamminga CA	40	Muller DJ	16
Caligiuri MP	39	Zai CC	16
Lohr JB	39	Citrome L	16
Kulkarni SK	37	Ivanova SA	14
Barnes TRE	37	Soares JC	14
Glazer WM	36	Tiwari AK	14
Caroff SN	34	Wang ZR	13
Chouinard G	33	Hashimoto K	12
Yassa R	33	Kosten TR	12
Sandyk R	32	Tan SP	12
Muller DJ	31	Blanchet PJ	11

### Most prolific journals

With a total of 271 publications, the *Journal of Clinical Psychiatry* was the most prolific journal overall, followed by the *American Journal of Psychiatry* and *Journal of Clinical Psychopharmacology*. During the last decade, the *Journal of Psychopharmacology*, *Schizophrenia Research* and *Journal of Clinical Psychiatry* were the most prolific. The top 25 journals (Table 3) accounted for 45.4% of all TD publications during the entire study period and 35.4% during the last decade.

**Table 3:** Most prolific journals in TD publications during the entire study period (1968–2021) and the last decade (2012–2021).

1968–2021			2012–2021		
Journal	No. of publications	%	Journal	No. of publications	%
Journal of Clinical Psychiatry	271	5.184	Journal of Clinical Psychopharmacology	32	3.226
American Journal of Psychiatry	249	4.763	Schizophrenia Research	31	3.125
Journal of Clinical Psychopharmacology	182	3.481	Journal of Clinical Psychiatry	28	2.823
British Journal of Psychiatry	142	2.716	Cochrane Database of Systematic Reviews	22	2.218
Psychopharmacology	140	2.678	Progress in Neuro- Psychopharmacology and Biological Psychiatry	21	2.117
Schizophrenia Research	137	2.621	Journal of The Neurological Sciences	17	1.714
Progress in Neuropsychopharmacology and Biological Psychiatry	123	2.353	Journal of Psychopharmacology	14	1.411
Biological Psychiatry	113	2.161	Parkinsonism Related Disorders	14	1.411
Movement Disorders	95	1.817	CNS Spectrums	13	1.310
Archives of General Psychiatry	87	1.664	Expert Opinion on Pharmacotherapy	12	1.210
Clinical Neuropharmacology	80	1.530	Movement Disorders	12	1.210
Psychopharmacology Bulletin	77	1.473	Neuropsychiatric Disease and Treatment	12	1.210
Acta Psychiatrica Scandinavica	76	1.454	PLOS One	12	1.210
Schizophrenia Bulletin	70	1.339	Behavioral Brain Research	11	1.109
Canadian Journal of Psychiatry Revue Canadienne De Psychiatrie	62	1.186	Pharmacology Biochemistry and Behavior	11	1.109
Psychiatry Research	60	1.148	Clinical Psychopharmacology and Neuroscience	10	1.008
Pharmacology Biochemistry and Behavior	56	1.071	Journal of Psychiatric Research	10	1.008
CNS Drugs	53	1.014	BMC Psychiatry	9	0.907
New England Journal of Medicine	50	0.956	Expert Review of Neurotherapeutics	9	0.907
International Clinical Psychopharmacology	49	0.937	Journal of Child and Adolescent Psychopharmacology	9	0.907
European Journal Of Pharmacology	43	0.822	Pharmacogenomics	9	0.907
Human Psychopharmacology Clinical and Experimental	42	0.803	Psychiatry Research	9	0.907
Journal of Psychopharmacology	39	0.746	Clinical Neuropharmacology	8	0.806
Pharmacopsychiatry	39	0.746	Human Psychopharmacology Clinical and Experimental	8	0.806
Cochrane Database of Systematic Reviews	38	0.727	Neuroscience Letters	8	0.806

### Most prolific institutions

With 322 publications (6.25%), US Department of Veterans’ Affairs was the most prolific affiliation related to TD research overall; however, its contribution has dropped down to 2.3% during the last decade (Table 4). The leading contributing institution in the last decade was the University of Toronto. Over the entire study period, the majority of the top 25 institutions (*n* = 19) were located in the USA, whereas 4 were in Canada, and 2 in England. In the last decade, only 13 of the top 25 were from the USA, while 2 universities from the Netherlands, and one each from China, Japan, France, Germany and Spain made the list.

**Table 4:** Most prolific affiliations associated with TD publications during the entire study period (1968–2021) and the last decade (2012–2021).

1968–2021			2012–2021		
Affiliation	No. of publications	%	Affiliation	No. of publications	%
US Department of Veterans Affairs	322	6.159	University of Toronto	65	6.552
Veterans Health Administration VHA	320	6.121	Baylor College of Medicine	60	6.048
University of California System	258	4.935	Center for Addiction Mental Health Canada	49	4.940
University of Toronto	194	3.711	Northwell Health	46	4.595
Northwell Health	154	2.945	University of California System	41	4.133
Center for Addiction Mental Health Canada	143	2.735	University of London	36	3.629
Harvard University	138	2.640	Peking University	34	3.427
National Institutes of Health NIH USA	134	2.563	Columbia University	26	2.621
University of London	122	2.334	Neurocrine Biosciences	25	2.520
NIH National Institute of Mental Health NIMH	107	2.047	University of Texas System	25	2.520
University of California San Diego	106	2.028	Chiba University	24	2.419
Pennsylvania Commonwealth System of Higher Education	105	2.008	Institut National de la Sante et de la Recherche Medicale Inserm	23	2.319
Baylor College of Medicine	103	1.970	US Department of Veterans Affairs	23	2.319
Yale University	100	1.913	Veterans Health Administration VHA	23	2.319
Columbia University	94	1.798	King’s College London	22	2.218
Yeshiva University	91	1.741	Universite De Montreal	21	2.117
University of Pittsburgh	89	1.702	University of Texas Health Science Center Houston	21	2.117
King’s College London	87	1.664	Maastricht University	20	2.016
University of California Los Angeles	84	1.607	University of Groningen	20	2.016
McGill University	81	1.549	University of Pennsylvania	20	2.016
Universite De Montreal	75	1.435	Northwestern University	19	1.915
Albert Einstein College of Medicine	70	1.339	State University System of Florida	19	1.915
New York University	69	1.320	Charite Universitatsmedizin Berlin	18	1.815
University of Texas System	68	1.301	Ciber Centro de Investigacion Biomedica en Red	18	1.815
University of North Carolina	65	1.243	Feinberg School of Medicine	18	1.815

WoS has conducted a unification process over the past few years, where institutions’ variant names and parent/child relationships are mapped and linked to a preferred institutional name. However, this unification process in WoS appeared to be still incomplete, and therefore, non-unified variants were merged manually where detected.

### Most prolific countries and collaborative networks

**Figure 2:**
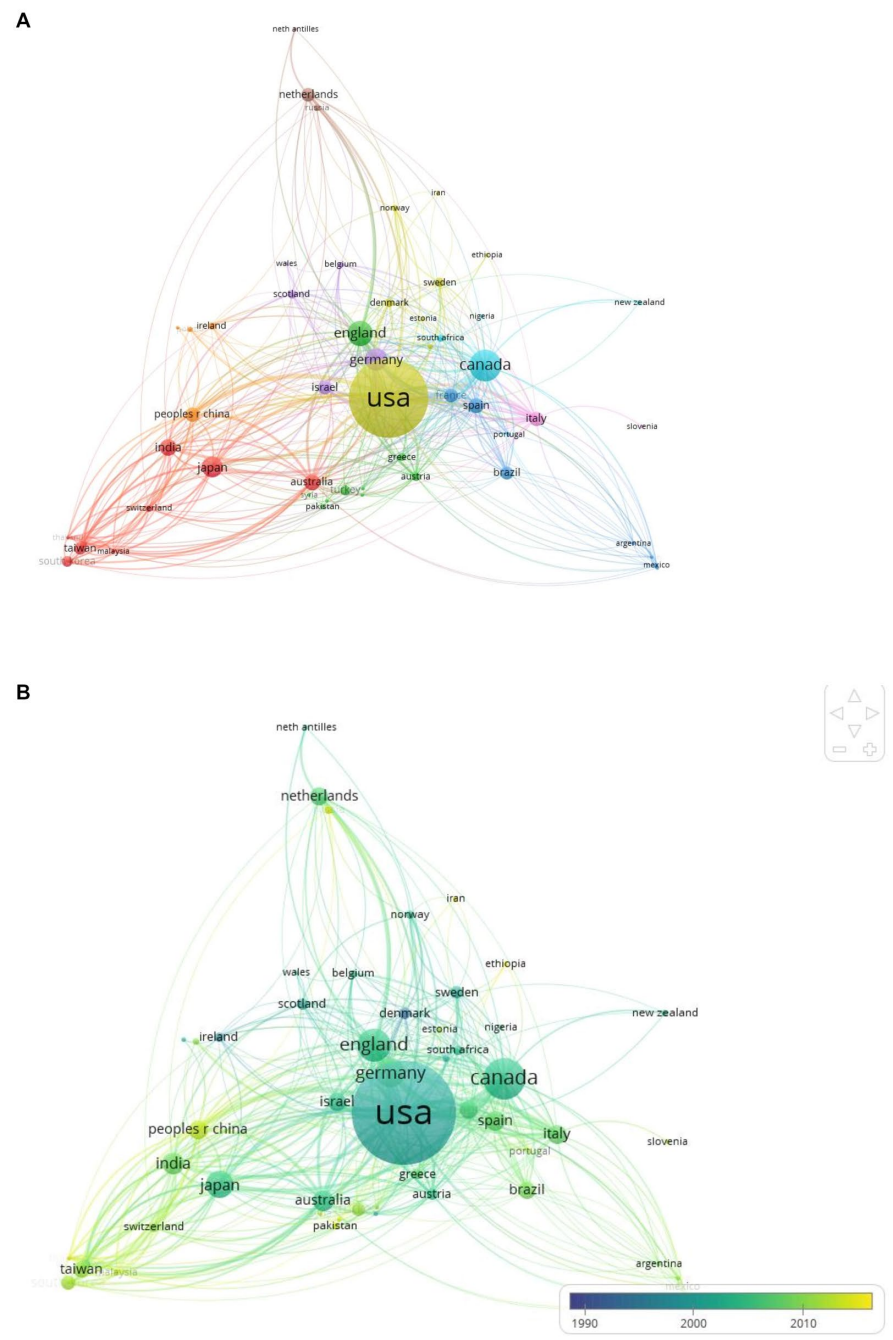
**(A)** Country co-authorship network visualization. Colors represent country clusters. Node size represents the number of publications in each country, and the thickness of lines connecting nodes indicates the strength of collaborations. **(B)** Overlay visualization displaying temporal trends. Yellow and yellow-green colored nodes indicate countries with more recent research activity.

The country with the highest TD research output was the USA, contributing to 44.3% of the literature overall, and 39% in the last decade (Table 5). The USA was followed by Canada, England, and Germany. Contributions from China have increased during the last decade (from 2.3 to 7.0%). Collaborations between countries visualized using VOSviewer based on co-authorship (Figure 2A) revealed that the USA had the greatest amount of links with other countries [total link strength (TLS) =616], followed by Canada (TLS =274) and England (TLS =238). Co-authorship among countries formed nine clusters: among them, Asian countries, Spanish-speaking countries, Mediterranean countries, and Scandinavian countries appeared to form separate clusters.

**Table 5:** Most prolific countries producing TD publications during the entire study period (1968–2021) and the last decade (2012–2021).

1968–2021			2012–2021		
Country	No. of publications	%	Country	No. of publications	%
USA	2,314	44.262	USA	387	39.012
Canada	478	9.143	Canada	117	11.794
England	361	6.905	England	84	8.468
Germany	283	5.413	Germany	71	7.157
Japan	244	4.667	People’s Republic of China	69	6.956
India	159	3.041	Japan	59	5.948
Australia	155	2.965	India	46	4.637
Italy	141	2.697	Italy	45	4.536
Spain	134	2.563	Australia	43	4.335
Netherlands	124	2.372	Netherlands	38	3.831
People’s Republic of China	122	2.334	Brazil	37	3.730
France	120	2.295	France	37	3.730
Israel	112	2.142	Spain	35	3.528
Brazil	107	2.047	Taiwan	31	3.125
Taiwan	92	1.760	Turkey	29	2.923
Denmark	79	1.511	South Korea	28	2.823
Sweden	75	1.435	Russia	22	2.218
South Korea	73	1.396	Switzerland	20	2.016
Turkey	71	1.358	Israel	19	1.915
Austria	63	1.205	Pakistan	15	1.512
Ireland	61	1.167	Poland	10	1.008
Scotland	61	1.167	Austria	9	0.907
South Africa	46	0.880	Saudi Arabia	9	0.907
Greece	45	0.861	Sweden	9	0.907
Switzerland	42	0.803	Scotland	8	0.806

Overlay visualization (Figure 2B) revealed temporal trends in publication activity across countries. Of note, several Asian (China, Malaysia, Thailand, South Korea) and Middle Eastern countries (Saudi Arabia) showed relatively recent surges in research activity.

#### Clusters of research and the evolution of knowledge on TD (1968–2021)

**Figure 3:**
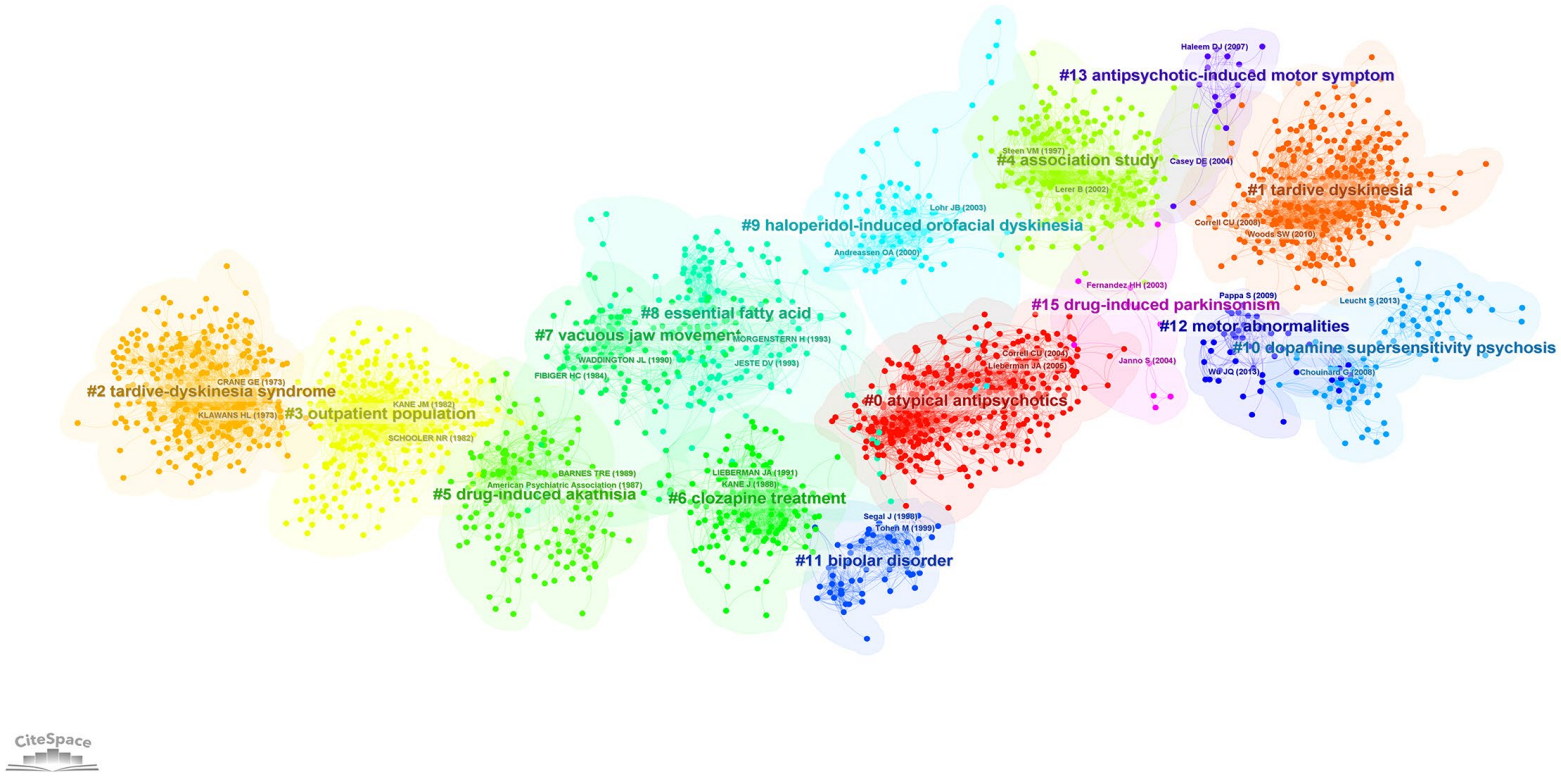
Co-citation network showing major research themes in tardive dyskinesia between 1968 and 2021, visualized using CiteSpace. Co-citation is the frequency of two publications being cited together. Colors represent research themes/clusters. The spatial arrangement of clusters represents the structural and temporal relationship among them. In this network, older clusters appear on the left and newer clusters on the right.

Co-citation analysis using CiteSpace revealed the major themes of research on TD (in the form of clusters) and their structural and temporal connections. Co-citation analysis of the 5,228 publications in the period of 1968–2021 identified 15 clusters (Figure 3), each labeled using title words. There was good quality in clustering configurations (weighted mean silhouette value = 0.89), and satisfactory clarity of network decomposition (modularity Q = 0.765).

**Figure 4:**
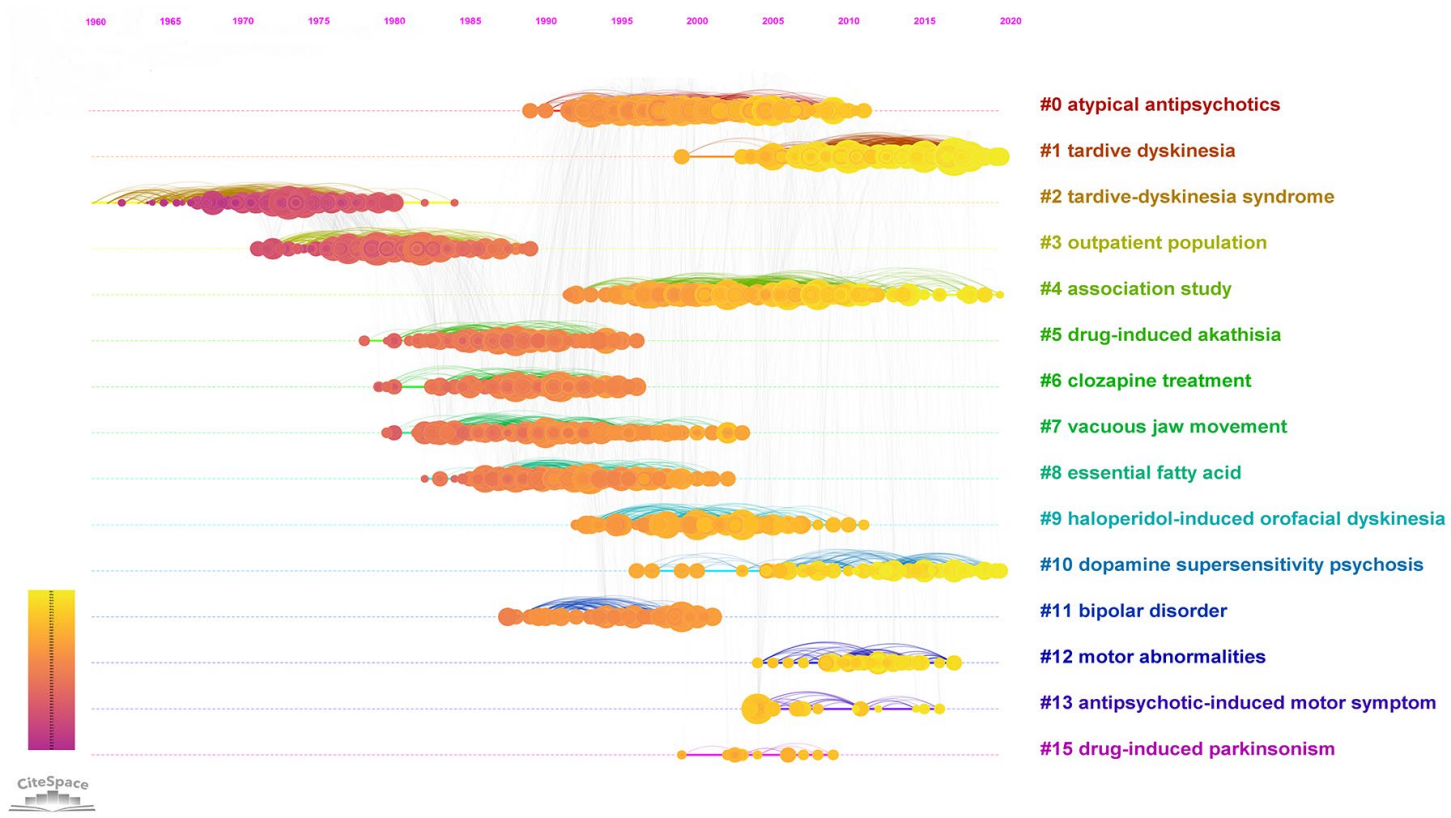
Timeline view of the 1968–2021 co-citation network. In the nodes, yellow represents newer knowledge, and purple represents older knowledge.

The temporal progression of knowledge across these clusters of research is illustrated in a Supplementary Video S1. A timeline view of the clusters also shows the evolution of scientific knowledge chronologically (Figure 4).

**Figure 5:**
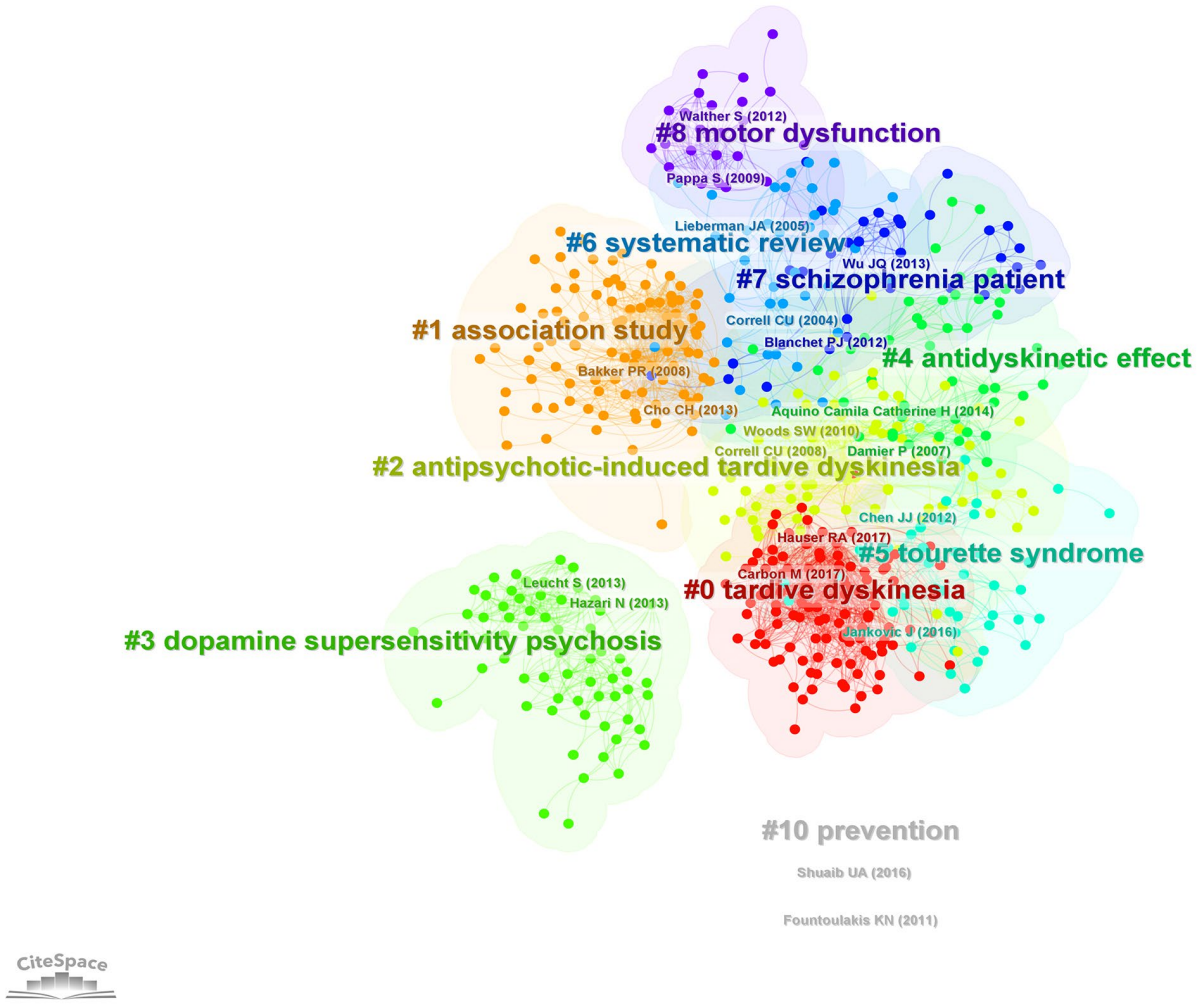
Co-citation network showing major research themes in the last decade (2012–2021) visualized using CiteSpace. Co-citation is the frequency of two publications being cited together. Colors represent research themes/clusters. The network is arranged based on structural and temporal relationships among the clusters.

**Figure 6:**
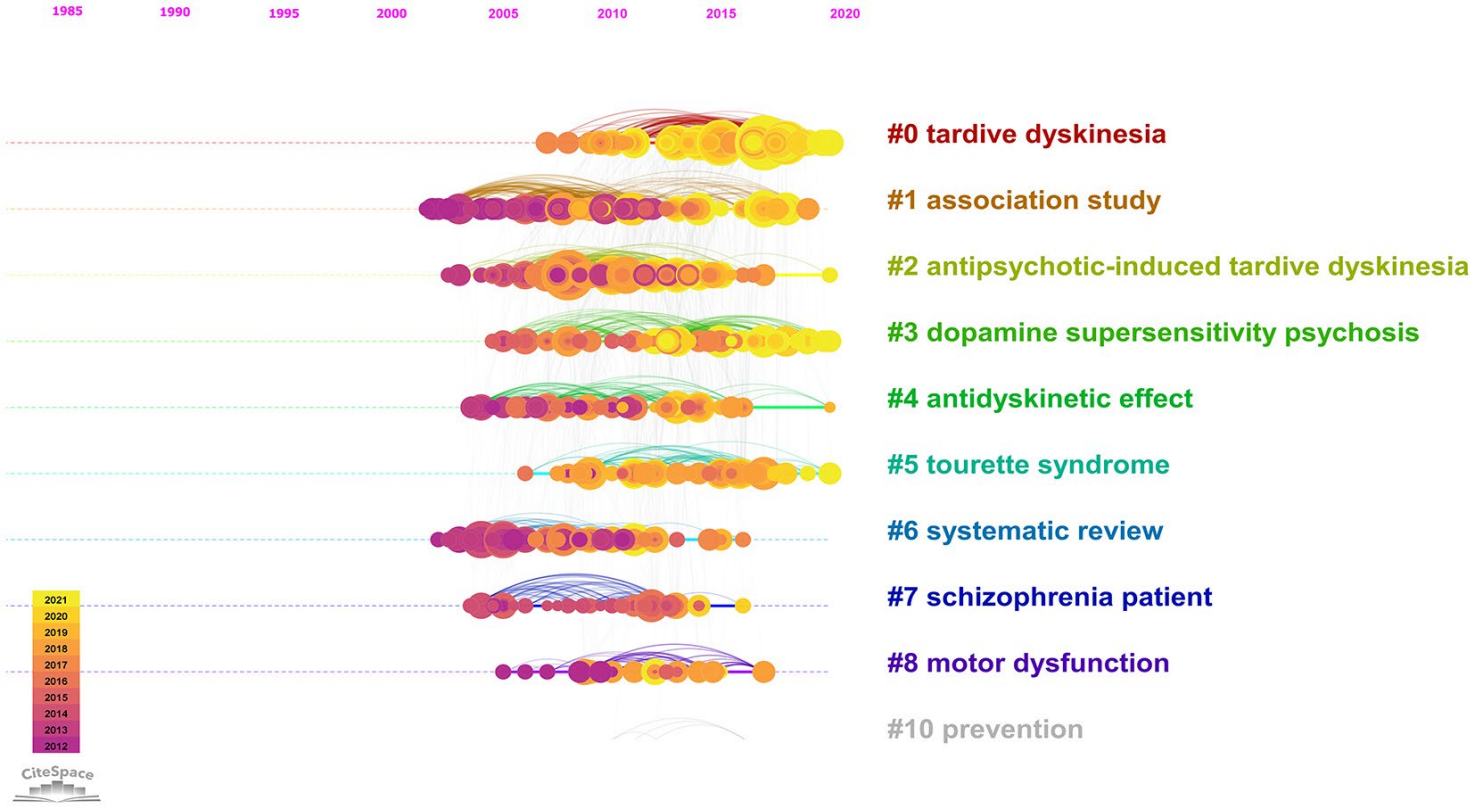
Timeline view of the 2012–2021 co-citation network. In the nodes, yellow represents newer knowledge, and purple represents older knowledge.

In Figure 3, the older knowledge clusters are at the left side of the visualized network, and the newer clusters are at the right-side end. A separate co-citation network for the last decade only (2012–2021) was also explored (Figures 5, 6).

## Historical developments in TD research

### Earliest publications on TD

The earliest publication indexed in WoS to include the term “tardive dyskinesia” in the title, abstract or keywords, was a review on TD by Crane (1968) (23). This article reviewed data from 21 publications describing TD in a total of approximately 500 patients. In this article, Crane cited the seminal work by Faurbye et al. (4) who coined the term “tardive dyskinesia” (4). Inspection of the bibliographic data for the article by Faurbye (4) revealed that TD was not mentioned in the title and there was no abstract available for this document. Crane (23) initially cited Sigwald et al. (24) as the first to report the syndrome, but in an addendum, gave credit to the earlier report from 1957 by Schonecker (3, 24).

### Early characterization of the TD syndrome

The oldest and the third largest cluster in the network (Figure 3), which commenced its activity in the 1960s (mean year =1971; cluster size = 309), was named “tardive dyskinesia syndrome.” This cluster represents the initial scholarly attempts to characterize the TD syndrome and its pharmacological aspects. The publication with the highest burstness within this cluster (burstness = 66.5) was an article on the pharmacology of TD by Klawans (1973) (25). A few early studies on therapeutic approaches for TD by Kazamatsuri et al. (1973, 1972) were identified as influential publications from this cluster (26, 27).

### Early epidemiological studies

The next cluster in chronological order (mean year = 1980) was named “outpatient population.” This cluster was characterized by early studies on TD epidemiology, and seems to have derived its name from studies conducted among “outpatient populations” (28, 29). Schooler and Kane’s (31) article which proposed criteria for diagnosing TD showed the highest citation burst within this cluster (30). The most influential publications from this cluster based on the sigma metric were a review by Kane and Smith (31) on the prevalence and risk factors of TD, and a study by Smith and Baldessarini (32) on the correlation between age and TD prevalence (31, 32) (Table 6).

**Table 6:** Top 25 most influential publications (based on sigma^a^ values) within TD literature.

Authors (year)	Title	Cluster	Sigma^a^	Centrality^b^	Burstness^c^
Correll and Schenk (33)	Tardive dyskinesia and new antipsychotics	#1 tardive dyskinesia	8887.36	0.13	75.46
Waddington (34)	Spontaneous orofacial movements induced in rodents by very long-term neuroleptic drug administration: phenomenology, pathophysiology and putative relationship to tardive dyskinesia	#9 vacuous jaw movement	349.24	0.16	39.87
Chouinard et al. (35)	A Canadian multicenter placebo-controlled study of fixed doses of risperidone and haloperidol in the treatment of chronic schizophrenic patients	#0 atypical antipsychotics	178.43	0.14	38.69
Kane et al. (36)	Integrating incidence and prevalence of tardive dyskinesia	#3 outpatient population	28.46	0.14	25.32
Kane et al. (36)	Clozapine for the treatment-resistant schizophrenic. A double-blind comparison with chlorpromazine	#6 clozapine treatment	25.17	0.05	71.24
Smith and Balderssarini (32)	Changes in prevalence, severity, and recovery in tardive dyskinesia with age	#3 outpatient population	18.18	0.07	42.45
Lieberman et al. (38)	Effectiveness of Antipsychotic Drugs in Patients with Chronic Schizophrenia	#0 atypical antipsychotics	10.71	0.03	83.39
Geddes et al. (39)	Atypical antipsychotics in the treatment of schizophrenia: systematic overview and meta-regression analysis	#0 atypical antipsychotics	9.49	0.08	30.11
Beasley et al. (40)	Olanzapine versus placebo and haloperidol: acute phase results of the North American double-blind olanzapine trial	#0 atypical antipsychotics	8.46	0.06	36.4
Casey (41)	Tardive dyskinesia and atypical antipsychotic drugs	#0 atypical antipsychotics	7.26	0.13	16.42
Bhidayasiri (42)	Evidence-based guideline: treatment of tardive syndromes: report of the Guideline Development Subcommittee of the American Academy of Neurology	#1 tardive dyskinesia	7.19	0.04	45.4
Yassa and Jeste (43)	Gender Differences in Tardive Dyskinesia: A Critical Review of the Literature	#8 essential fatty acid	6.9	0.07	27.2
Jones et al. (44)	Randomized controlled trial of the effect on Quality of Life of second- vs. first-generation antipsychotic drugs in schizophrenia: Cost Utility of the Latest Antipsychotic Drugs in Schizophrenia Study (CUtLASS 1)	#0 atypical antipsychotics	6.47	0.06	31.5
Waddington et al. (45)	Spontaneous orofacial dyskinesia and dopaminergic function in rats after 6 months of neuroleptic treatment	#9 vacuous jaw movement	6.12	0.09	20.5
Woods et al. (46)	Incidence of tardive dyskinesia with atypical versus conventional antipsychotic medications: a prospective cohort study	#1 tardive dyskinesia	5.84	0.04	50.6
Lerer et al. (47)	Pharmacogenetics of tardive dyskinesia: combined analysis of 780 patients supports association with dopamine D3 receptor gene Ser9Gly polymorphism	#4 association study	5.81	0.04	42.7
Jeste et al. (48)	Lower Incidence of Tardive Dyskinesia with Risperidone Compared with Haloperidol in Older Patients	#0 atypical antipsychotics	5.02	0.04	38.2
Kane and Smith (31)	Tardive dyskinesia: prevalence and risk factors, 1959 to 1979	#3 outpatient population	4.69	0.02	79.4
Kapur and Seeman (49)	Does Fast Dissociation From the Dopamine D_2_ Receptor Explain the Action of Atypical Antipsychotics?: A New Hypothesis	#0 atypical antipsychotics	4.07	0.05	28
Lohr et al. (50)	Oxidative mechanisms and tardive dyskinesia	#7 haloperidol induced orofacial dyskinesia	3.85	0.04	37
Tsai et al. (51)	Markers of Glutamatergic Neurotransmission and Oxidative Stress Associated With Tardive Dyskinesia	#7 haloperidol induced orofacial dyskinesia	3.67	0.04	29.7
Chouinard et al. (52)	Tardive dyskinesia and antiparkinsonian medication	#3 outpatient population	3.59	0.09	14.9
Andreassen and Jørgensen (53)	Neurotoxicity associated with neuroleptic-induced oral dyskinesias in rats. Implications for tardive dyskinesia?	#7 haloperidol induced orofacial dyskinesia	3.21	0.03	36.7
Correll et al. (54)	Lower risk for tardive dyskinesia associated with second-generation antipsychotics: a systematic review of 1-year studies	#0 atypical antipsychotics	3.2	0.01	93.4
Gunne et al. (55)	Association with persistent neuroleptic-induced dyskinesia of regional changes in brain GABA synthesis	#9 vacuous jaw movement	3.07	0.05	23.5

^a^Sigma is a composite indicator of both structural and temporal properties of a node in the network, and is computed as (centrality + 1)^burstness^. ^b^Centrality indicates the extent to which a node lies in the middle of a path connecting different nodes. ^c^Burstness indicates a specific duration in which an abrupt change in citation frequency takes place.

### Cognitive dysfunction and TD

The next cluster to emerge (mean year = 1987) was rather inaptly named “drug induced akathisia” due to the presence of many articles on akathisia citing this cluster. Inspection of the contents of this cluster revealed that the more relevant theme was the relationship between cognitive dysfunction and TD. In fact, another algorithm (latent semantic indexing) named this cluster as “cognitive dysfunction.” The most influential article on the latter theme (sigma = 1.42, burstness = 25.8) was a study by Waddington and colleagues (1987) on the association between cognitive dysfunction, negative symptoms, and tardive dyskinesia (56).

As seen in Figure 3, starting around the late 1980s, the progression of scientific inquiry appears to have proceeded along two parallel pathways: One pathway includes the clusters “vacuous jaw movements,” “essential fatty acids” and “haloperidol-induced orofacial dyskinesia,” and the other pathway includes the clusters “clozapine treatment,” “bipolar disorder” and “atypical antipsychotics.” The contents of these clusters will be explored next.

### Animal models of TD

The cluster named “vacuous jaw movements,” which become active during the late 1980s (mean year = 1988), represents animal studies conducted to elucidate the mechanisms underlying drug-induced movement disorders. It appears to have derived its name from studies on “vacuous jaw movements,” a form of chewing movements observed in rodents following administration of certain pharmacologic agents, e.g., dopamine antagonists. The most influential publication in this respect was a review article by Waddington (1990) that critically evaluated the evidence for a putative rodent model for TD and identified inconsistencies in the extant literature (34). This article had the highest centrality value of all publications in the overall network (centrality = 0.16), signifying a pivotal point within the TD literature.

### Oxidative mechanisms

The cluster named “essential fatty acids” which became active during the early 1990s (mean year = 1992) revealed a research interest directed toward antioxidants and free radical pathology in relation to schizophrenia and TD. The most relevant article citing this cluster was a review by Reddy and Yao (1996) on free radical pathology in schizophrenia (57). The closely linked cluster named “haloperidol-induced orofacial dyskinesia” (mean year = 1998), with notable structural overlap with the “essential fatty acids” cluster, seems to have extended the scientific inquiry into the neurochemical basis of TD, with continuing inputs derived from animal studies. The publications with the highest burstness from this cluster were on oxidative mechanisms of TD (49), neurotoxicity associated with neuroleptic-induced oral dyskinesias in rats (53) and pathophysiology and animal models of TD (41).

### Clozapine and TD

A cluster named “clozapine treatment” (mean year = 1990) reflected the rise in the use of clozapine as a treatment for schizophrenia around that time and its potential impact on TD literature. A pioneering clinical trial by Kane and colleagues (37) which evaluated the effectiveness of clozapine versus chlorpromazine for treatment-resistant schizophrenia has experienced a strong citation burst from 1989 to 1998 (burstness = 71.2), and was the most influential publication (sigma = 25.17) within this cluster (36). Another article with high burstness within this cluster was a review on the effect of clozapine on TD by Lieberman et al. (38); this article reviewed eight studies on the effects of clozapine on TD and found that approximately 43% of patients improved after clozapine treatment (58).

### TD in bipolar disorder

A relatively small cluster named “bipolar disorder” had become active during the 1990s (mean year = 1995, cluster size = 60). This cluster represents the increased attention directed toward the use of antipsychotics for the treatment of bipolar disorder and the consequent concerns for TD. The publication with the highest burst strength in this cluster was a placebo-controlled study that assessed the efficacy and safety of olanzapine for the treatment of acute mania (59). In this study, no significant difference in the risk of TD was found for olanzapine in comparison with a placebo.

### Clinical trials of atypical antipsychotics

The largest cluster in the network, with 330 publications, was named “atypical antipsychotics” (red cluster in Figure 3). This cluster became active in the early 1990s and continued its activity till around 2010, reflecting a surge of research on the efficacy and tolerability of atypical antipsychotics, and their relationship to TD. Among the most influential publications in this cluster were clinical trials comparing atypical antipsychotics with typical ones (35, 38, 40) and a meta-analysis (39) comparing the same. The publication with the strongest citation burst of all time was also part of this cluster: A systematic review of 1-year studies comparing the risk of TD for atypical antipsychotics against typical antipsychotics (54) showed a strong citation burst from 2005 to 2014 (burstness = 93.4). The article with the second highest centrality value (indicating a pivotal point) within the whole network, a Canadian placebo-controlled trial that compared risperidone and haloperidol for the treatment of schizophrenia (35) was also part of this cluster. This study had demonstrated a significant beneficial effect of risperidone for TD.

### Pharmacogenetics of TD

During the late 1990s, a relatively large cluster named “association study” had started to become active (cluster size = 236). It represents pharmacogenetic studies of TD. This cluster has been highly active in the early 2000s (mean year =2004), and its activity seems to have persisted till date. The most influential article within this cluster was a study on the association between TD and the dopamine D3 receptor gene Ser9Gly polymorphism (47); their findings supported a small but significant contribution of the DRD3 ser9gly polymorphism to TD susceptibility. A strong citation burst can be observed also for an earlier study on the dopamine D3-receptor gene variant (60). The second most influential publication in this cluster was a meta-analysis of pharmacogenetic interactions of TD (61).

## Recent trends

The more recent research interests related to TD are reflected by the clusters on the right side of the network shown in Figure 3. A timeline perspective of these clusters revealed four clusters with significant activity during the last decade (Figure 4). To further expand on recent trends, a separate co-citation network for publications from the last decade only (1,001 publications from 2012–2021) was also examined (Figures 5, 6). This latter network demonstrated 10 clusters, most of which corresponded to clusters in Figure 3 in terms of research themes. Notably, a cluster named “Tourette syndrome,” which was not observed in the 1968–2021 network, appeared in the 2012–2021 network. The cluster named “schizophrenia patient” in the 2012–2021 network appeared to be a continuation of interest in cognitive dysfunction in schizophrenia, with a greater focus on brain-derived nerve-growth factor and superoxide dismutase in the recent period.

**Figure 7:**
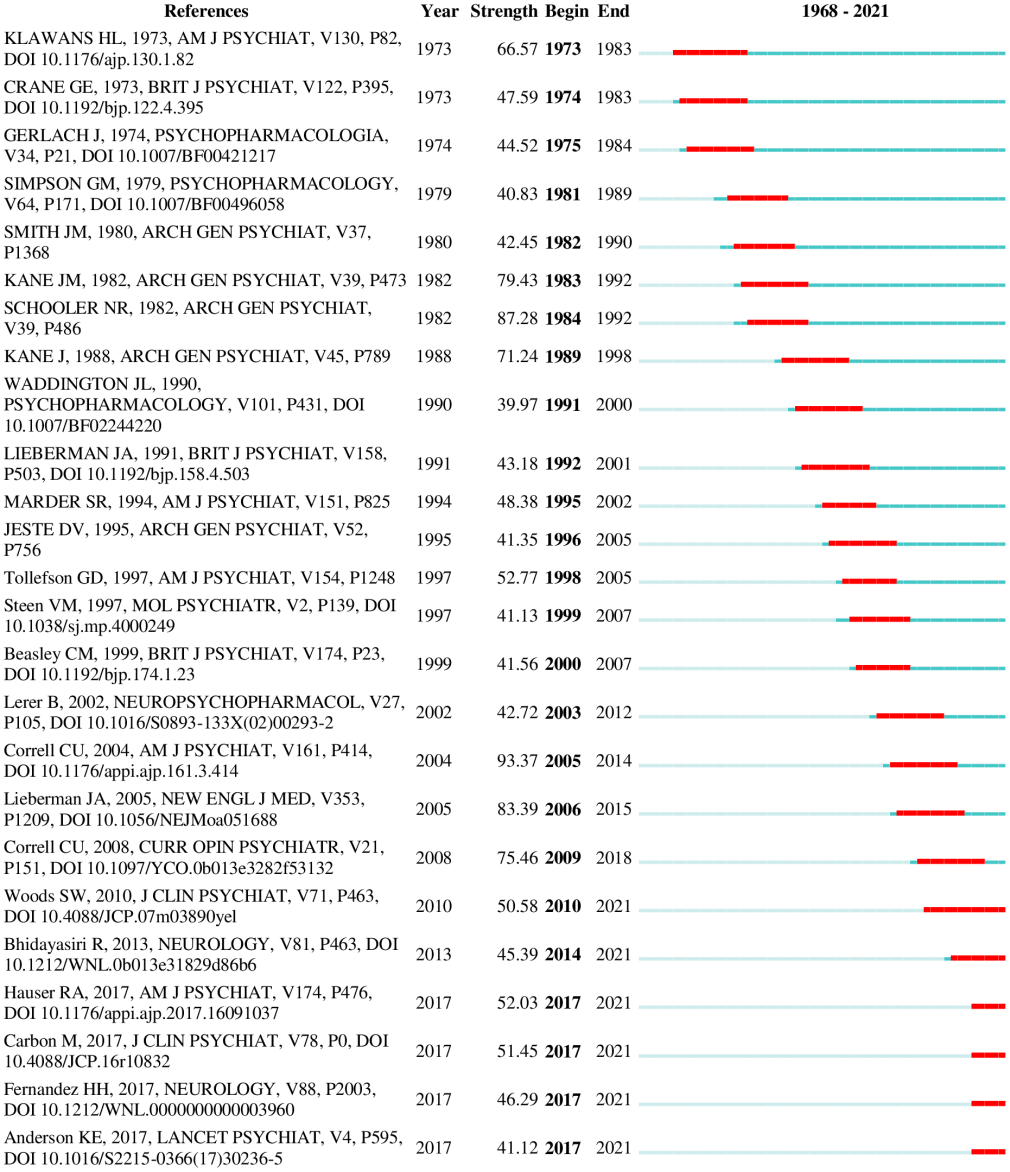
Top 25 references with the strongest citation bursts (1968–2021). A burst indicates a sudden surge in the use of a specific keyword during a specific period. The duration of burst is depicted by the red line appearing along the timeline. The last six studies show bursts still in progress.

**Figure 8:**
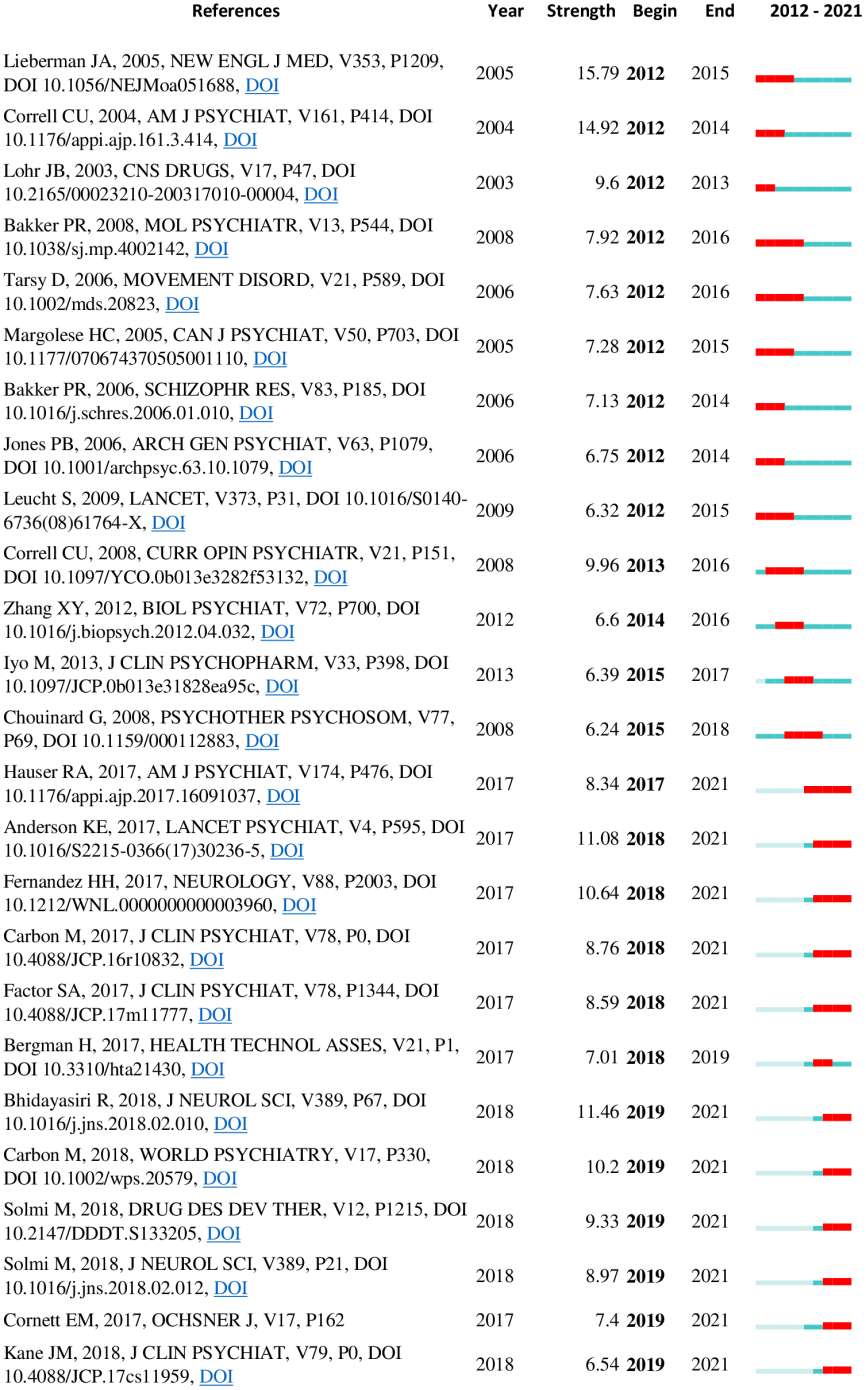
Top 25 references with the strongest citation bursts from the 2012–2021 co-citation network. A burst indicates a sudden surge in the use of a specific keyword during a specific period. Duration of citation burst is depicted by the red line appearing along the timeline. Some studies show bursts still in progress.

In addition to the co-citation networks and clusters, recent citation bursts and keyword trends also provided insight into recent trends. Some noteworthy citations bursts (Figure 7) from the recent years are mentioned under respective clusters below. The top 25 citation bursts from the 2012–2021 network were also examined separately (Figure 8) to identify notable bursts from the recent years, since older publications tend to have higher burst values overall.

### Serotonergic receptors and deep brain stimulation

A cluster named “antipsychotic induced motor symptom” (cluster size = 18, mean year = 2009) revealed an interest in the role of serotonergic receptors, particularly the 5HT_2C_ receptor, in antipsychotic-induced motor symptoms including TD. The most relevant article citing this cluster was a review on the role of serotonin in antidyskinetic effects of DBS (62). The most influential publication associated with this cluster (sigma =23.6) was a review by Casey (2004) where the author discusses the pathophysiology of antipsychotic-induced movement disorders, including the role of serotonergic receptors in mitigating motor effects (63). The “antidyskinetic effect” cluster from the 2012–2021 network corresponded with this theme (Figure 5), and additionally demonstrated an increasing focus on DBS as a treatment for TD.

### Primary motor abnormalities of schizophrenia

A cluster named “motor abnormalities” (cluster size = 49, mean year = 2011), which has been active during the last decade, examines the notion that primary motor abnormalities are part of a neurodysfunction intrinsic to the pathogenesis of schizophrenia. The most influential publication in this cluster (sigma = 12.8) was a systematic review on spontaneous movement disorders in antipsychotic-naive patients with first-episode psychoses (64). The most relevant citer of this cluster was a review (65) postulating motor dysfunction as an intermediate phenotype across schizophrenia and other psychotic disorders. The corresponding cluster from the 2012–2021 network was named as “motor dysfunction.”

### Dopamine supersensitivity psychosis

The concept of “dopamine supersensitive psychosis” appears to have attracted attention within the TD literature during the last decade, as reflected by the eponymous cluster (mean year = 2012). Influential articles in this cluster included an editorial discussing the relevance of DSP for atypical antipsychotics, and a review on the optimal extent of D2 receptor occupancy by antipsychotics for the treatment of DSP (66, 67). The most relevant article citing this cluster was a recent review on diagnostic considerations in DSP (68). A corresponding cluster with the same name can be seen in the 2012–2021 network.

### Recent epidemiological studies and advances in TD treatment

The most recent and the second largest cluster in the overall network (mean year = 2013, size = 309) was named “tardive dyskinesia.” Two clusters from the 2012–2021 network (“tardive dyskinesia” and “antipsychotic induced tardive dyskinesia”) corresponded with this theme. Inspection of this cluster identified two main lines of research interest: a renewed interest in the epidemiology of TD particularly in regard to atypical antipsychotics, and studies exploring new treatment options for TD, such as VMAT-2 inhibitors. The most influential publication in the whole network (Table 6) was identified from this cluster; this was a review by Correll and Schenk (2008) providing updated rates of TD with typical and atypical antipsychotics (33). Other influential publications on TD epidemiology included a more recent meta-analysis of TD prevalence in the period of atypical antipsychotic use (8) and a prospective cohort study investigating the incidence of TD with atypical versus conventional antipsychotic medications (46). Both these studies are currently experiencing strong citation bursts. Several publications on newer treatment options for TD are also showing currently active citation bursts; these include clinical trials of deutetrabenazine (69, 70) and valbenazine (71) as well as an evidence-based guideline formulated in 2013 by the American Academy of Neurology (42) for the treatment of tardive syndromes. Clinical trials on amantadine (72) and Vitamin B6 (73) were also included among influential publications. The most relevant publication citing this cluster was a recent review on advances in the pharmacology of tardive dyskinesia (74). A 2018 update (75) to the earlier guideline by the American Academy of Neurology was found to be the most influential publication from the last five years in the 2012–2021 co-citation network.

### Hyperkinetic movement disorders

A cluster named “Tourette syndrome” (cluster size = 43, mean year = 2013) emerged in the 2012–2021 network. This cluster represents the overlapping treatment options for hyperkinetic movement disorders, such as Tourette syndrome, Huntington’s chorea and TD. Particularly, an interest in the use of dopamine depleting agents, such as VMAT-2 inhibitors, for the treatment of hyperkinetic disorders other than TD was seen in this cluster. The most influential article on this theme was a 2016 review on the use of dopamine depleters in the treatment of hyperkinetic movement disorders (76), and the most important citer of this cluster was a 2018 review on treatment options for chorea (77).

### Keyword patterns and trends

**Figure 9:**
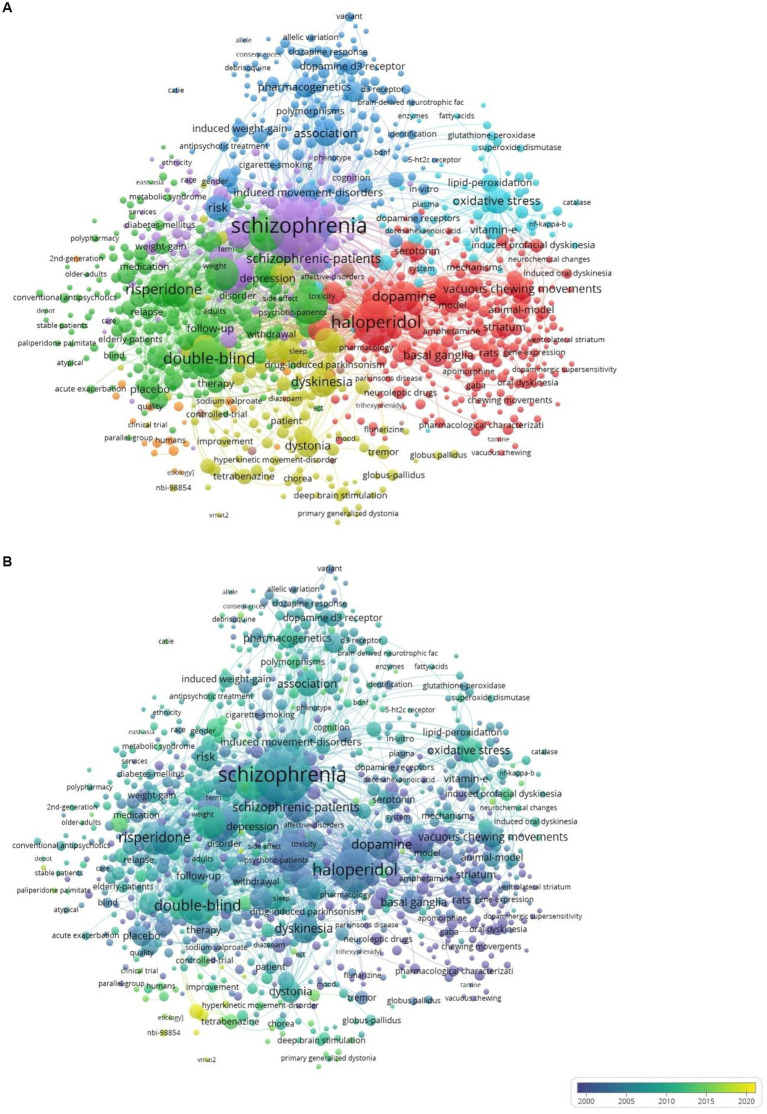
**(A)** Keyword co-occurrence network visualized using VOSViewer. Colors denote keyword clusters. Node size represents the number of times each keyword was used. The thickness of lines connecting keywords indicates how frequently they co-occurred. **(B)** Overlay visualization. Yellow and yellow-green colored nodes indicate keywords with more recent surges.

The keyword co-occurrence network for the top 1,000 keywords visualized using VOSViewer is shown in Figure 9A. The 10 most frequently co-occurring keywords (apart from tardive dyskinesia) were *schizophrenia, haloperidol, double-blind, clozapine, antipsychotics, atypical antipsychotics, risperidone, dopamine, prevalence* and *olanzapine*. Keywords were algorithmically grouped into 7 clusters. The main themes identified from these keyword clusters were animal models and neuroanatomical correlates (cluster #1, red, 278 items), efficacy and safety of antipsychotics (cluster #2, green, 274 items), pharmacogenetics (cluster #3, deep blue, 174 items), movement disorders and treatment options (cluster #4, yellow, 123 items), epidemiology (cluster #5, purple, 96 items) and oxidative processes (cluster #6, light blue, 68). The seventh and smallest cluster (orange, 28 items) appeared to be a conceptual overlap between cluster #2 and cluster #4.

The overlay visualization of the keyword network (Figure 9B) illustrates temporal trends in keyword occurrence. The most recent keywords (shown in yellow or yellow-green) were *VMAT 2 inhibitor* (mean year = 2019.5), *deutetrabenazine* (mean year = 2019.1), *valbenazine* (mean year = 2018.7), *nbi-98,854* (mean year = 2018.5), *dopamine supersensitivity psychosis* (mean year = 2017.6), and *tardive syndrome* (mean year = 2017.56).

Keyword occurrence bursts visualized using CiteSpace also revealed temporal trends. Data for keywords were available in WoS only from 1990 onwards. The 100 strongest keyword bursts from 1990 to 2021 are shown in Supplementary Figure S1. Some salient observations on keyword bursts include the following. Keywords related to animal studies (e.g., *rat rat brain*) had shown bursts during the 1990s. Many keywords related to neurochemistry (e.g., *receptor binding antagonist agonist D2 receptor D1 dopamine receptor*) also showed bursts during the same decade. The keywords *neuroleptics neuroleptic treatment neuroleptic drug* have been active during that period whereas the terms *antipsychotics* and *atypical antipsychotics* seem to have become active only beyond 2005. Some keywords related to neuroanatomy (*nucleus accumbens substantia nigra striatum* and *basal ganglia*) were also identified from the 1990s onward. Among the dopamine receptors D1 and D2 were cited in the early 1990s and D3 became active after 2000. Among names of antipsychotics *clozapine* showed a burst from 1995 to 2002; whereas risperidone (1999–2004) olanzapine (2001–2013) and quetiapine (2003–2010) appeared subsequently. Keywords related to pharmacogenetics such as *D3 receptor gene variant susceptibility* and *promoter region* showed bursts between 2000 and 2010. Bursts for some keywords related to metabolic side effects such as *diabetes mellitus* (2002–2008) and *weight gain* (2002–2010) indicated the concerns about concurrent metabolic effects of antipsychotics following the widespread use of atypical antipsychotics. Terms related to oxidative stress have shown bursts during the 1990s (alpha tocopherol vitamin E) 2000s (manganese superoxide dismutase nitric oxide) and the 2010s (oxidative stress lipid peroxidation). *Quality of life* showed a burst in 2005–2015 indicating an interest in the impact of TD on the quality of life of patients. Keywords related to treatment methods for TD have become active mostly after 2008. In this time period *botulinum toxin* (2008–2021) *deep brain stimulation* (2010–2021) *tetrabenazine* (2015–2021) *ginkgo biloba* (2015–2018) *deutetrabenazine* (2017–2021) and *nbi-98,854/valbenazine* (2017–2021) each showed keyword bursts. Other recent bursts with currently ongoing activity are noted for *bipolar disorder* (2019–2021) *major depressive disorder* (2017–2021) *double blind/controlled trial* (2018–2021) *movement disorder* (2015–2021) and *first episode* (2014–2021).

## Discussion

### Summary of the main findings

In this scientometric review, we have mapped the evolution of scientific knowledge about TD over a period of 54 years (1968–2021) using state-of-the-art scientometric mapping tools. Publication trends over the years as well as the most prolific authors, institutions and countries during the entire study period and during the last decade (2012–2021) were summarized. Research activity peaked in the 1990s, gradually declined after 2004, and showed a small increase again after 2015. Network of co-cited references revealed fifteen hotspots (or clusters) in research. Structural and temporal metrics identified the most important publications within the network; the most influential publication was a review by Correll and Schenk (2008) on TD rates for atypical versus typical antipsychotics (33).

Beginning in the 1960s, the first clusters represented a research trend that sought to characterize the clinical features, pharmacological basis, and epidemiology of TD. This trend continued into the 1980s, and then the corpus of literature diverged into two directions by the latter part of the decade and two parallel trends became active in the 1990s; one trend attempted to elucidate the etiology of TD with studies on animal models and oxidative mechanisms, while another trend was based on clinical trials comparing atypical against typical antipsychotics. Etiologic studies seemed to be linked to the more recent knowledge base by a cluster of pharmacogenetic studies. The most recent clusters represented interest in the role of serotonergic receptors and DBS, DSP, primary motor abnormalities of schizophrenia, hyperkinetic disorders, advances in the treatment of TD, and updated epidemiologic evidence.

### Trends in TD research

At the peak of research activity during the 1990s, researchers had been focused on studying the pathophysiology of TD on the one hand, and the risk of TD with atypical antipsychotics, on the other. The rising availability of new technology would have facilitated pathophysiological studies during this period, whereas the widespread introduction of atypical antipsychotics in the 1990s and the possibility of a lower risk of TD with these newer antipsychotics would have stimulated research interest in the latter. Notably, quality of life in relationship to TD has only emerged as an issue in the 2005–2015 period, likely due to the lack of a TD-specific quality of life and functionality scale (78–80) despite increasing focus on patient reported outcomes and quality of life in the area of psychiatry at large. Since the turn of the century, research interest in TD had started to dwindle, possibly because of the assurances provided by influential studies published around that time (54) indicating a lower risk of TD with newer antipsychotics. However, a subsequent update on TD risk for atypical antipsychotics in 2008, which was identified as the most important publication, showed that the incidence of TD with atypical antipsychotics was higher than previously reported. Despite this evidence, interest in TD continued to decline until 2014. Another reason for the dwindling interest in TD may be the shift of focus from motor side effects to metabolic side effects of antipsychotics, a prominent trend that was noted after 2000 in a previous scientometric paper (22). Similarly, in our analysis of keywords, *weight gain* and *diabetes mellitus* showed bursts between 2000 and 2010. The apparent resurgence of interest after 2015 would have been consequent to the promising results seen for VMAT-2 inhibitors as a treatment method for TD and other hyperkinetic disorders (76, 81, 82) substantiated by the Food and Drug Authority (FDA) approval of two VMAT-2 inhibitors – valbenazine and deutetrabenazine- in 2017 for the treatment of TD. Starting in 2017, some key publications on valbenazine (71) and deutetrabenazine (69, 70) have been displaying strong citation bursts, while the same keywords started to show bursts since 2017. A keyword burst for tetrabenazine (an older VMAT inhibitor), which started in 2015, is also ongoing. It is interesting to note that, in spite of some influential publications from the early 1970s (26, 27) that studied tetrabenazine as a treatment for TD, the interest in VMAT-2 inhibitors had remained dormant for more than four decades.

Scholars have debated the differentiation between spontaneous dyskinesias and TD for many decades (1). However, interest in this area seems to have been revived recently. According to the most important publication from this theme, spontaneous dyskinesias were reported in 9% of antipsychotic-naive patients with first-episode psychoses (64). There appears to be an emerging interest in studying motor dysfunction as an intermediate phenotype of psychotic illnesses (65).

DSP has also garnered attention during recent years. It has been posited that dopamine hypersensitivity resulting from long-term treatment with dopamine receptor blocking agents may lead to tolerance to the antipsychotic effects leading to an unstable psychotic state associated with TD. Such destabilization has also been observed as part of breakthrough psychosis even during assured treatment with long-acting antipsychotics (83, 84). Although cumulative antipsychotic dose was not a consistent risk factor for breakthrough psychosis, TD was indeed a risk factor (85). Consequently, DSP has been associated with treatment resistance in schizophrenia, and some important publications on this theme (66, 67) were identified by this study.

Pharmacogenetics has become a thriving research area in many fields of medicine over the last few decades, with the increased availability of genetic testing facilities. Pharmacogenetic studies in relation to TD have also become active in the late 1990s, and the influence of this trend of research is still visible. Many potential risk genes for TD, particularly the dopamine receptor gene variants, have been investigated (47, 60, 61). However, the evidence appears to be inconclusive, and the contributions from the studied gene variants to TD risk have been small. In the future, if risk genes with better predictive value for TD are identified, genetic testing might allow personalization of antipsychotic selection, thereby minimizing the incidence of TD.

### Uses and practical implications of the findings

Scientometrics (or bibliometrics) is a useful method to efficiently review a large and rapidly expanding body of literature to identify its trends and hotspots (11, 18). Knowledge of the most prominent authors and institutions would be useful to many groups of people. Researchers searching for collaborations and postgraduate research candidates looking for mentors or supervisors would benefit from the knowledge of prominent authors and institutions working on TD. In our study, we mentioned the most influential publications related to each research hotspot; this knowledge can be useful for anyone looking for literature on these topics, and to understand the evolution of knowledge on TD in general. The top journals in TD publications presented here can be useful for those who are searching for literature on TD and for researchers who are choosing a journal to submit their TD-related papers to. The prominent keywords under different themes identified within the literature can be useful in creating search queries in database searches and in listing relevant keywords in manuscripts.

The majority of the literature on TD was based on Western data. Although Japan, China and India have made important contributions to the field, and a recent increase in publications from Asian countries was visible, more research on TD among non-Western populations would be warranted since genetic and cultural variations may limit the applicability of Western findings to other populations.

### Strengths and limitations

To the best of our knowledge, this is the first study to conduct a comprehensive bibliometric analysis and mapping of the extant literature on TD. Since the term “tardive dyskinesia” is a unique term with little possibility of being used outside its current sense, most of the articles selected from the database using this keyword would have some relevance for TD. Our choice of scientometric tools allowed us to visually represent trends and hotspots of research, which facilitates the reader’s understanding of the findings. In contrast to previous narrative reviews which had described the history of TD research (1, 5), our review has a more comprehensive coverage of the literature and was guided by objective indicators, such as structural and temporal metrics. The co-citation network and the clusters had good decomposition and cluster configurations, supporting the validity of these findings.

Our study also has several limitations. The bibliographic data were obtained from the WoS only. Although WoS is one of the largest databases, there may have been publications in other databases not indexed by WoS, leading to limited coverage (86). Currently, there is no reliable method to de-duplicate combined datasets based on multiple databases for co-citation analysis. WoS was chosen as its data are generally considered the most suitable for co-citation analysis (22).

In general, bibliometric analyses do not systematically screen all included articles for their relevance to the topic. Although we included publications with the term “tardive dyskinesia” in the title, abstract or keywords, there would have been publications without direct relevance to TD, and also, we may have missed some publications related to TD. Similarly, although we identified the most influential and pivotal publications using statistical indicators, the quality and risk of bias in these articles were not ascertained. Studies which are cited more frequently are not necessarily of high quality; studies that receive criticism within the scientific community for poor quality can also receive more citations. Citation bias can occur due to a variety of reasons. For example, positive study outcomes, the authority of the authors, and higher journal impact factor are associated with greater likelihood of being cited (87). Since the co-citation networks are generated using citation data, these biases can influence the results. In addition, publication bias is widespread and may have influenced these findings (88). Industry sponsorship may have also influenced the productivity in TD research and operated as a source of publication bias. It is possible that repetition and duplication of publication reflect marketing objectives rather than scientific merit in some areas, such as antipsychotic and anti-TD drug development. However, the extent of these biases could not be assessed in the present bibliometric method. Finally, more recent trends may not have been captured by co-cited networks, as the most recent publications have fewer citations.

## Conclusion

This bibliometric study visualized the evolution of scientific knowledge on TD over more than five decades and identified some emerging trends. The most prolific authors, institutions and countries were identified. Early scientific interest in the 1970s-1980s dealt with clinical features and epidemiology of TD, followed by pathophysiological studies and clinical trials comparing the risk of TD with dopamine receptor blocking agents in the 1990s. After a peak of research interest in the 1990s, interest in TD gradually declined, but a small resurgence of interest was observed following the FDA approval of two VMAT-2 inhibitors in 2017 as active treatment options for TD. Recent research interests included the role of serotonergic receptors in TD, DBS as a treatment, DSP, primary motor abnormalities of schizophrenia, hyperkinetic disorders, recent updates on TD treatment and epidemiology. These bibliometric findings can be useful for researchers to find relevant literature when writing scientific articles, choosing appropriate journals, finding collaborators or mentors for research, and to understand the historical developments and current trends in TD research.

## Data availability statement

The original contributions presented in the study are included in the article/Supplementary materials, further inquiries can be directed to the corresponding author.

## Author contributions

AB and CC contributed to the study conceptualization and design. AB performed the literature search, analysis of data, and drafted the manuscript. CC contributed to the interpretation of data. All authors contributed to and have approved the final manuscript.

## Conflict of interest

CC has been a consultant and/or advisor to or has received honoraria from: AbbVie, Acadia, Alkermes, Allergan, Angelini, Aristo, Boehringer-Ingelheim, Cardio Diagnostics, Cerevel, CNX Therapeutics, Compass Pathways, Darnitsa, Gedeon Richter, Hikma, Holmusk, IntraCellular Therapies, Janssen/J&J, Karuna, LB Pharma, Lundbeck, MedAvante-ProPhase, MedInCell, Merck, Mindpax, Mitsubishi Tanabe Pharma, Mylan, Neurocrine, Newron, Noven, Otsuka, Pharmabrain, PPD Biotech, Recordati, Relmada, Reviva, Rovi, Seqirus, SK Life Science, Sunovion, Sun Pharma, Supernus, Takeda, Teva, and Viatris. He provided expert testimony for Janssen and Otsuka. He served on a Data Safety Monitoring Board for Lundbeck, Relmada, Reviva, Rovi, Supernus, and Teva. He has received grant support from Janssen and Takeda. He received royalties from UpToDate and is also a stock option holder of Cardio Diagnostics, Mindpax, LB Pharma and Quantic.

The remaining author declares that the research was conducted in the absence of any commercial or financial relationships that could be construed as a potential conflict of interest.

## Publisher’s note

All claims expressed in this article are solely those of the authors and do not necessarily represent those of their affiliated organizations, or those of the publisher, the editors and the reviewers. Any product that may be evaluated in this article, or claim that may be made by its manufacturer, is not guaranteed or endorsed by the publisher.
